# Beyond GLMs: A Generative Mixture Modeling Approach to Neural System Identification

**DOI:** 10.1371/journal.pcbi.1003356

**Published:** 2013-11-21

**Authors:** Lucas Theis, Andrè Maia Chagas, Daniel Arnstein, Cornelius Schwarz, Matthias Bethge

**Affiliations:** 1 Werner Reichardt Centre for Integrative Neuroscience, Tübingen, Germany; 2 Graduate School of Neural Information Processing, University of Tübingen, Tübingen, Germany; 3 Hertie Institute for Clinical Brain Research, Tübingen, Germany; 4 Graduate School of Neural and Behavioural Sciences, University of Tübingen, Tübingen, Germany; 5 Bernstein Center for Computational Neuroscience, Tübingen, Germany; 6 Max Planck Institute for Biological Cybernetics, Tübingen, Germany; Philipps-University Marburg, Germany

## Abstract

*Generalized linear models* (GLMs) represent a popular choice for the probabilistic characterization of neural spike responses. While GLMs are attractive for their computational tractability, they also impose strong assumptions and thus only allow for a limited range of stimulus-response relationships to be discovered. Alternative approaches exist that make only very weak assumptions but scale poorly to high-dimensional stimulus spaces. Here we seek an approach which can gracefully interpolate between the two extremes. We extend two frequently used special cases of the GLM—a linear and a quadratic model—by assuming that the spike-triggered and non-spike-triggered distributions can be adequately represented using Gaussian mixtures. Because we derive the model from a generative perspective, its components are easy to interpret as they correspond to, for example, the spike-triggered distribution and the interspike interval distribution. The model is able to capture complex dependencies on high-dimensional stimuli with far fewer parameters than other approaches such as histogram-based methods. The added flexibility comes at the cost of a non-concave log-likelihood. We show that in practice this does not have to be an issue and the mixture-based model is able to outperform generalized linear and quadratic models.

## Author Summary

An essential goal of sensory systems neuroscience is to characterize the functional relationship between neural responses and external stimuli. Of particular interest are the nonlinear response properties of single cells. Inherently linear approaches such as generalized linear modeling can nevertheless be used to fit nonlinear behavior by choosing an appropriate feature space for the stimulus. This requires, however, that one has already obtained a good understanding of a cells nonlinear properties, whereas more flexible approaches are necessary for the characterization of unexpected nonlinear behavior. In this work, we present a generalization of some frequently used generalized linear models which enables us to automatically extract complex stimulus-response relationships from recorded data. We show that our model can lead to substantial quantitative and qualitative improvements over generalized linear and quadratic models, which we illustrate on the example of primary afferents of the rat whisker system.

## Introduction

To account for the stochasticity inherent to neural responses, single cells as well as populations of cells are often characterized in terms of a probabilistic model. A popular choice for this task are generalized linear models (GLMs) and related approaches [1]–[6]. These models can often be chosen such that the corresponding maximum likelihood problem is a convex optimization problem where a global optimum can be found. This guarantee comes at a price, as GLMs tightly constrain the computations which can be performed on the input. More complex computations can nevertheless be implemented by choosing a nonlinear feature representation of the input which is then fed into the linear model. In practice, however, it is typically very challenging to select the appropriate feature space because it presupposes a deeper understanding of the cell's nonlinear behavior or unfeasibly large amounts of data.

Several approaches have been suggested to overcome the limitations of the generalized linear model. A natural extension is given by *generalized quadratic models* [7]–[9]. While a quadratic model represents a true generalization of a linear model, it can also be viewed as a linear model with a quadratric extension of the feature space (and, depending on the parametrization, some additional constraints on the parameters). Consequently, it shares similar limitations. A linear combination of quadratic features might still fail to represent the kind of stimulus properties a neuron responds to, but going to higher-dimensional general-purpose feature spaces quickly leads to overfitting. The number of parameters which need to be estimated grows linearly with the stimulus dimensionality in a linear model, quadratically in a quadratic model, and correspondingly faster if one uses a feature space of higher order.

An alternative approach is offered by nonparametric methods such as *maximally informative dimensions* (MID) [10]. Here, one first seeks a projection of the stimulus onto a lower-dimensional subspace such that as much information as possible is retained about the presence or absence of a spike. Afterwards, histograms are used to map out the nonlinear dependence of the neuron on the projected stimulus. This approach has the advantage that it can, at least in principle, capture arbitrary dependencies on the stimulus. However, the number of parameters that need to be estimated grows exponentially with the dimensionality of the stimulus subspace. This limits the approach to cells which are selective for only a few stimulus dimensions, although nonlinear extensions of this approach exist [11].

Here, we explore a different tradeoff. We derive a much more flexible neuron model for single cells which can, at least in principle, approximate arbitrary dependencies on the stimulus. The model can be viewed as generalizing generalized linear and quadratic models, but in contrast to quadratic models cannot easily be reduced to a GLM by choosing a different representation of the stimulus. Nonlinear stimulus features are directly learned from the data by maximizing the model's likelihood and do not need to be hand picked. The number of parameters of the model still grows only quadratically with the dimensionality of the stimulus, and the complexity of the model can be tuned to take into account the cell's complexity and the amount of data available. We demonstrate that optimizing this model is feasible in practice and can lead to a better fit than either generalized linear or quadratic models.

## Methods

In the following—after briefly reviewing generalized linear and quadratic models—we introduce a new model for single cell responses and discuss its properties.

### Ethics statement

All experimental and surgical procedures were carried out in accordance with the policy on the use of animals in neuroscience research of the Society for Neuroscience and the German law.

### Generalized linear and quadratic models

In a GLM it is assumed that the output 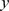 conditioned on some input 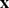 is distributed according to an exponential family and that the expected output is given by
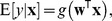
where 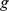 is an invertible nonlinearity. Parameters of the model are the weights 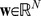 and potentially additional parameters of the exponential family. In the following, we will assume that 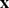 is a representation of the stimulus and 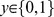 indicates the presence or absence of a spike.

A special case of the GLM applicable to our problem is, for instance, the linear-nonlinear-Bernoulli (LNB) model, where the exponential family is given by the Bernoulli distribution. As nonlinearity we might choose the sigmoidal logistic function,
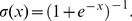
(1)In the following, we will derive this linear model from a generative modeling point of view. This will help to motivate and see the connections to the extension presented later.

Let us consider the distribution over the stimulus 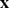 conditioned on 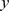. If 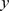 equals 1, this distribution corresponds to the spike-triggered distribution. If 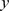 equals 0, we will call it the *non-spike-triggered distribution*. At least for the moment, let us assume that both distributions are Gaussian, that is,
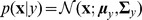
with means 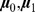 and covariances 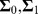. Bayes' rule allows us to turn these assumptions into a probabilistic model of the neuron's behavior,
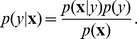
Using a few simple calculations, the probability of observing a spike, or *firing rate*, can be seen to be
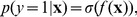
(2)where
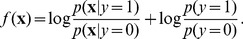
(3)Using our assumption of Gaussianity, this reduces to
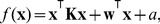
(4)where we have performed the reparametrization
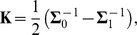


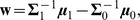
and the bias term is given by
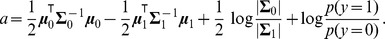
If the spike-triggered and non-spike-triggered covariances are assumed identical, the quadratic term vanishes and we obtain the linear-nonlinear-Bernoulli model from above. Without this assumption, we are left with a quadratic model [7]–[9].

The unconstrained quadratic model is equivalent to a GLM with a quadratic extension of the feature space, since
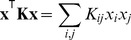
(5)is linear in the parameters 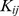. In practice, 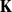 is often replaced by a low-rank approximation 
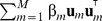
 [7]–[9], [12], where 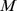 controls the rank. The quadratic form in this case is given by
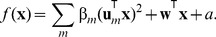
(6)When choosing this parametrization, the optimization is no longer a convex problem [9] and the model no longer a GLM. In the following, we will use “quadratic model” only to refer to the unconstrained version—a GLM with a quadratic feature space—and “linear model” to refer to the GLM without quadratic features.

### Spike-triggered mixture model

The generative point of view immediately suggests generalizations by loosening the assumptions of Gaussian distributed spike-triggered and non-spike-triggered stimuli. In the following, we consider mixtures of Gaussians as possible candidates,
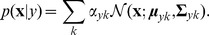
Mixture models provide a good compromise between the assumptions of the tightly constrained generalized linear models and nonparametric approaches such as histograms. By plugging the mixture distributions into Equation 3, we obtain a new neuron model whose complexity can be controlled by adjusting the number of mixture components. We dub this model the *spike-triggered mixture model* (STM).

In the same manner that we have derived a model for the neuron's dependency on the stimulus, we can incorporate dependence on other features as well. Let 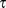 be the time past since the neuron fired its last spike. Using Bayes' rule, we obtain
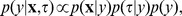
where here we have made the additional assumption that 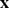 and 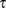 are independent given 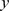. This assumption is also known as the *naive Bayes* assumption and is often employed in classification. It has empirically been observed that naive Bayes classifiers often perform well even when the assumption of independence is not met [13], [14].

**Figure 1:**
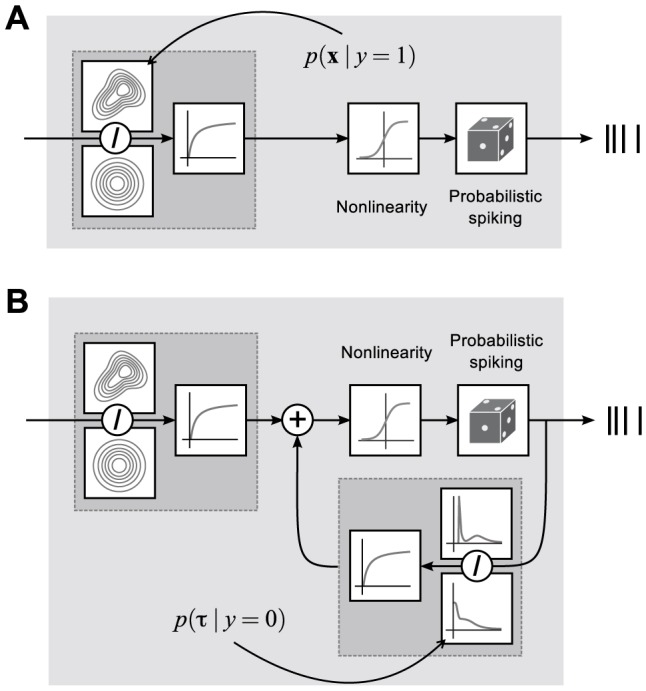
Illustration of the spike-triggered mixture model (STM). **A**. A sigmoidal nonlinearity is applied to a log-likelihood ratio of two mixtures of Gaussians to determine the firing rate of the model, which is then used to generate spikes. **B**. By making a naive Bayes assumption, additional information and measurements such as interspike interval distributions can easily be incorporated into the model in the form of additional log-likelihood ratios.

Taken together, the input to the sigmoid nonlinearity (Equation 2) is given by
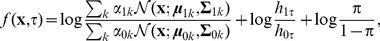
(7)where 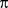 represents the prior probability of observing a spike and we have used histograms 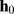 and 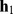 to represent the interval distributions, 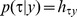 (Figure 1). Note that if we do not constrain the parameters, there are several redundancies in this parametrization. For example, we can multiply both 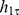 and 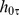 by a common factor without changing the model's predictions. If we reparametrize the model to get rid of redundancies and in addition assume that one mixture component is enough to represent the non-spike-triggered distribution, the input to the sigmoid takes the much simpler form
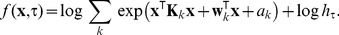
(8)The assumption of Gaussian distributed non-spike-triggered stimuli is sensible, for instance, if an *a priori* Gaussian distributed stimulus is used to drive the neuron and the width of each bin of the spike train is small such that the posterior probability of observing a spike is generally also small, since in this case
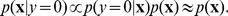
The spike history dependent term on the right-hand side of Equation 8 can also be written in terms of a linear filter,
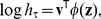
where 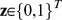 represents the spike history, and 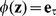 is the unit vector with zeros everywhere except at the position of the most recent spike. That is, the only difference to a linear model with history dependent term 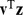 is that here all but one spike are suppressed by 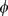. In our experiments, we found that the two forms of spike history dependency worked equally well for most cells.

It is instructive to compare Equation 8 with Equation 4. While the quadratic model can be cast into the form of a linear model with a quadratic feature space, this is in general not possible for the STM. The function 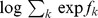 is also known as *soft maximum*, since it can be viewed as a smooth approximation to the maximum of the 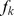. Our model is thus effectively taking the maximum of the responses of a number of quadratic models. Also note that the number of parameters is only a constant times the number of parameters of the quadratic model, which means it still grows only quadratically in the number of stimulus dimensions. But the number of parameters can be reduced further, as discussed in the next section.

### Reducing the number of parameters

Assuming a single non-spike-triggered mixture component as in Equation 8 and ignoring the spike history for the moment, the number of parameters of the STM grows as 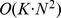, where 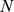 is the stimulus dimensionality and 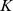 is the number of mixture components. This growth might still be impractical where 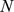 is large or the amount of available data is small, as is often the case with neural data.

To reduce the number of parameters, we can employ the same trick as for the quadratic model and replace the matrices 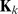 by low-rank approximations (Equation 6). If we additionally share features 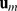 between the different components, we obtain
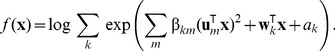
(9)The number of parameters now grows as 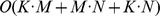, where 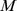 is the number of quadratic features 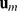 contributing 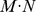 parameters, 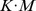 is the number of coefficients 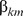, and 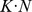 is the number of parameters added by the linear features 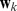. That is, for fixed 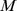 and 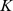, the number of parameters is linear in the number of stimulus dimensions. We will refer to this variant of the model as the *factored STM*.

### Experimental setup

**Figure 2:**
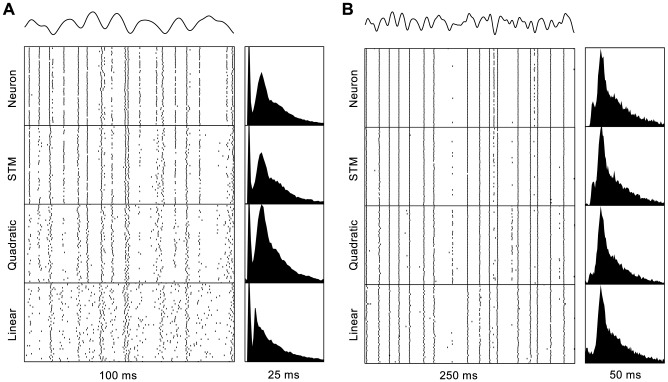
Spike trains generated by real and model neurons. Stimuli corresponding to the spike trains are shown at the top. The first row below the stimulus shows spike trains and interspike interval distributions generated by one *slowly adapting* (**A**) and one *rapidly adapting* cell (**B**) of the rat's whisker system. The two cells shown are the SA cell and RA cell where the quantitative improvement in performance gained by using an STM over a quadratic model was largest.

We tested our model on spike trains obtained from 18 whisker-sensitive trigeminal ganglion cells of adult Sprague-Dawley rats. Recordings were made with a single electrode (sampling frequency: 20 kHz, bandpass filter: 300–5000 Hz). Manual stimulation was used to identify which whisker the neuron innervated as well as the approximate preferred direction of the whisker, after which the whisker was placed inside a plastic tube driven by a metal stimulator arm. The stimulator arm was programmed to deliver low-pass filtered (100 Hz) Gaussian white noise stimulation along the neuron's preferred movement direction. Stimulation was divided into 50 *unfrozen trials* in which the stimulation sequence varied between trials, and 50 *frozen trials* in which a Gaussian white noise sequence was generated for the first trial only and then repeated for each subsequent trial. Spikes were extracted offline on the basis of waveform shape and all cells were categorized as either *slowly adapting* (SA) or *rapidly adapting* (RA). Example spike trains of two cells for frozen stimuli are shown in Figure 2.

We extracted 10 ms windows from the stimulus and reduced their dimensionality by keeping the first 10 principal components (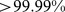 explained variance). We also extracted 25 ms of the spike history preceding each bin of the spike train. The dimensionality of the spike history was reduced to 100 by binning spikes into 100 equally sized bins of 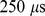 width (no bin contained more than 1 spike). We then removed all but the most recent spike from the spike history and used this as input to all models. A linear projection of this vector is equivalent to the form of spike history dependency in Equation 8.

Filters of generalized linear models were first trained assuming a sigmoid nonlinearity. Together with a Bernoulli output distribution, this guarantees a concave log-likelihood such that an optimal solution can be found. Afterwards, we replaced the sigmoid nonlinearity with a more flexible nonlinearity consisting of a sum of Gaussian blobs,
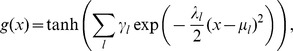
where the hyperbolic tangent ensures that the predicted probability of a spike does not exceed 1. We jointly optimized the parameters of this nonlinearity and the linear filter by alternately maximizing the average log-likelihood of the linear-nonlinear model using limited-memory BFGS [15], a standard quasi-Newton method (see Text S1 of the supporting information for gradients of the parameters). In a final step, we used a nonparametric histogram estimate (150 bins) to map out the nonlinearity. Through this multi-step procedure we tried to maximize the chances of finding a linear-nonlinear description of a neuron's behavior where one exists. Note that strictly speaking, this model is no longer a generalized linear model (since the nonlinearities used are not invertible and the nonlinearities' parameters are optimized). Quadratic models were optimized using the same procedure after extending the input by quadratic features.

The parameters of the STM (Equation 7) were initialized by estimating the spike-triggered, non-spike-triggered, and interspike interval distributions. Mixtures of Gaussians were fitted using standard expectation maximization [14], [16] and interval distributions were estimated using histograms. While naive Bayes classifiers often already work well, it can be beneficial to directly optimize the conditional log-likelihood [17]. After initializing the parameters, we thus discriminatively finetuned the parameters using BFGS [18]. We found that this indeed helped to substantially improve the performance where the model depended on both the stimulus and the spike history.

We used between three and five components for the spike-triggered distribution and one and two components for the non-spike-triggered distribution, which was found to work well in preliminary runs on a different but related dataset with similar stimuli. Using two non-spike-triggered components increased the stability of the optimization for some cells. Finally, factored STMs were trained discriminatively using limited-memory BFGS with randomly initialized parameters.

All models were trained on the 50 unfrozen trials and performance was evaluated based on the 50 frozen trials.

## Results

We qualitatively and quantitatively compare the performance of the generalized linear, quadratic and spike-triggered mixture model (STM) for different cells and find in both cases that the STM can lead to substantial improvements.

### Qualitative comparison

**Figure 3:**
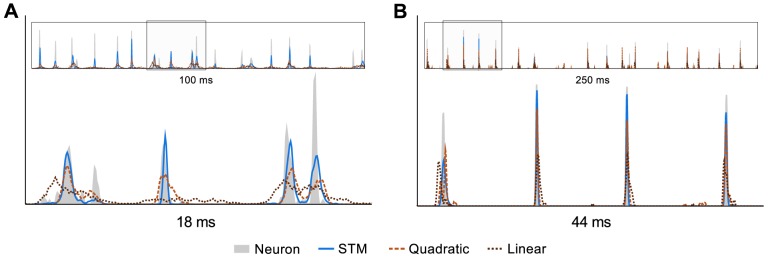
Peristimulus time histograms. The insets show peristimulus time histograms (PSTHs) corresponding to the spike trains in Figure 2 (best viewed on a computer screen). PSTHs were estimated from 50 trials for real cells, 1000 trials for model cells, and smoothed using a Gaussian kernel. The kernel width was chosen automatically (see text) except for the zoomed-in excerpt of the PSTH in **B**, where for better visibility we used a slightly wider kernel (FWHM: 0.15 ms). The variance explained (R^2^) by the generalized linear model, quadratic model and STM was 0.15, 0.26, and 0.47 (**A**), and 0.19, 0.41, and 0.5 (**B**), respectively.

Figure 2 shows spike trains generated by the different models when fitted to one SA cell and one RA cell. The trial-to-trial variability of the responses of most cells in the dataset is quite low. This behavior is well captured by the STM, while the responses of the generalized linear and quadratic models generally seem to be noisier. This difference is more pronounced for SA cells than for RA cells, where all models appear to give a reasonably good fit. Corresponding peristimulus time histograms (PSTHs) can be seen in Figure 3 (details on how the PSTHs were computed are given in the next section).

**Figure 4:**
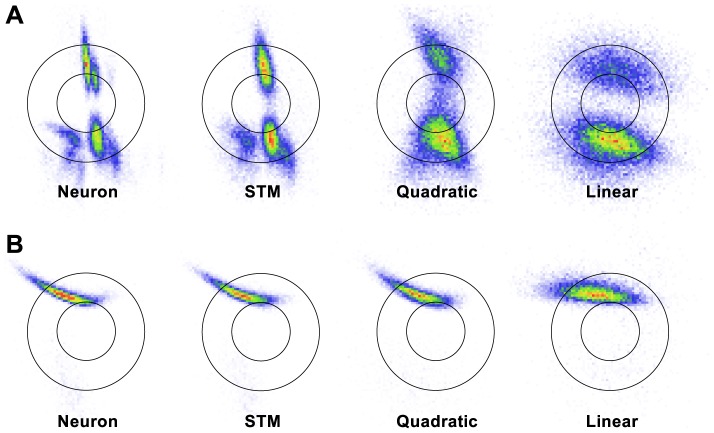
Spike-triggered distributions of real and model neurons. The plots show spike-triggered histograms of positions 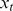 (horizontal axis) and velocities 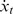 (vertical axis) of the stimulus for the same neurons displayed in Figure 2, that is, for one SA cell (**A**) and one RA cell (**B**). Stimuli were measured 1.5 ms before a spike occured. Note that these are not the stimuli the STM was trained on, which were 10-dimensional. Solid lines indicate one and two standard deviations of the Gaussian prior stimulus distribution.

Similar conclusions can be drawn by looking at spike-triggered distributions (Figure 4). Ensembles of spike-triggered positions 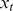 and velocities 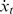 of the time-varying stimulus suggest a complex dependency of the responses on the stimulus for at least some cells. Note, however, that even a linear neuron can produce non-Gaussian spike-triggered distributions when the stimulus is correlated over time and the cell's firing depends on its history of generated spikes. Also note that while here we show 2-dimensional spike-triggered distributions, the input to the models was a 10-dimensional stimulus (and a 100-dimensional spike history), as described above.

To get a better intuition for the degree of nonlinearity of a cell, we can compare the cell's spike-triggered distribution with the spike-triggered distribution of the best matching linear model. In the given examples, the linear model is unable to reproduce the spike-triggered distributions of the cells displayed in Figure 4. For the SA cell, even the quadratic model fails to reproduce many of the features of the neuron's spike-triggered distribution, while the STM's behavior much more closely resembles that of the real cell.

### Quantitative comparison

To quantify the performance of the different models, we estimate the *cross-entropy* or negative log-likelihood,
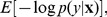
(10)where the expectation is taken over stimuli 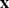 and spikes 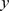 generated by the real neuron. We estimate this quantity using 50 frozen trials not used during training of the model. The cross-entropy is a natural measure for comparing different models, as it is the measure which is optimized during maximum likelihood estimation of the parameters, and many other system-identification approaches such as spike-triggered averaging can often be viewed as performing maximum likelihood or penalized maximum likelihood learning [19].

The cross-entropy can be used to lower-bound the mutual information between stimuli and spikes,
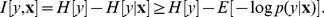
The better a model distribution 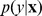 approximates a cell's behavior, the smaller the difference will be between the lower bound and the true information transmitted by the cell. Note that this mutual information only quantifies the information carried by one bin of the spike train while we are generally more interested in the information carried by an entire spike train, 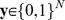.

The spike train's mutual information with the stimulus can be decomposed as follows
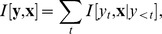
where 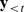 denotes the history of spikes preceding time 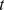. To correctly quantify the mutual information between the spike train and the stimulus, it is thus imporant to take spike history effects into account. If we also use the fact that a neuron's firing will only be affected by the stimulus preceding a spike, 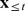, we get
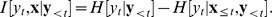
for the mutual information of the spike train per time bin. Dividing by the bin width yields an information rate (measured in bits per second or similar). Estimating this quantity requires two models: one for approximating the distribution 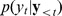 and one for approximating 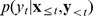. A model for the former can take the form of Equation 8 but with the stimulus-dependent terms dropped.

**Figure 5:**
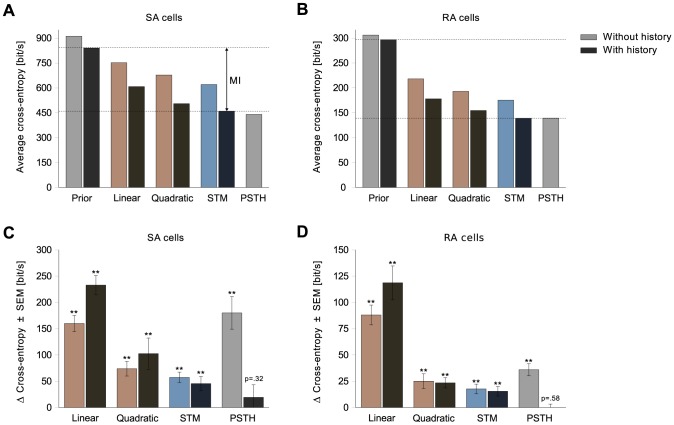
Quantitative model comparison. Linear, quadratic and spike-triggered mixture models (STM) were evaluated on 8 slowly adapting cells (**A**) and 10 rapidly adapting cells (**B**). The performance of each model is measured in terms of the cross-entropy (negative log-likelihood) averaged over all cells (smaller is better). Light bars correspond to models which ignore the spike history, dark bars correspond to models which explicitly take the spike history into account. By subtracting the cross-entropy from the estimated entropy of the spike trains (“Prior”), an estimate of mutual information (MI) between stimuli and spike trains is obtained. The bars in **C** and **D** show (from left to right) the differences in performance between the linear model and the prior, the quadratic model and the linear model, and the STM and the quadratic model (with and without spike history dependency, respectively). The two right most bars show the improvement of the PSTH over the STM with and without spike history dependency.

Results averaged over all recorded SA cells (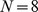) and all RA cells (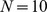) are given in Figure 5. The average improvement of the STM over the quadratic model is 45.40 bit/s for SA cells and 15.48 bit/s for RA cells (for models taking into account spike history). The improvement for the cell with the largest difference to the quadratic model is 95.15 bit/s for SA cells and 43.05 bit/s for RA cells (the cells displayed in Figures 2 to 4). The firing rates of these two neurons were 117 Hz and 52.6 Hz, respectively, so that both numbers roughly correspond to 0.8 bit/spike improvement. These improvements correspond to the amount of information carried by the cells that would have been missed if a quadratic model was used to estimate mutual information instead of an STM. The average differences between the quadratic and the linear model, and the STM and the quadratic model (with and without including spike history) were all significant (one-tailed Wilcoxon signed-rank test, 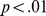; Figure 5C and D).

In addition to comparing different models, we can also compute and compare our model's performance to the cross-entropy of a PSTH, which has also been called *oracle model* [20]. We computed PSTHs by convolving the average response to the frozen stimulus with a Gaussian kernel. We took all but one trial to compute the PSTH and the remaining trial for prediction. That is, the probability of a spike at time 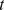 in trial 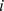 was predicted to be
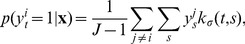
(11)where 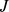 is the number of trials and 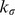 is a normalized Gaussian kernel of width 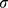,
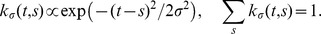
For spike counts larger than 1, the same approach could be taken by using the right-hand side of Equation 11 as the rate parameter of a Poisson distribution. We found it was necessary to add a small offset to the PSTH to achieve good results. Both the offset and the kernel width were automatically chosen from a prespecified set of parameters to minimize the cross-entropy averaged over all trials. That is, for each individual cell, we chose the kernel width with the best prediction performance. The optimal kernel widths were found to be around 0.12 ms and 0.09 ms (full width at half maximum, FWHM) for the SA and the RA cell displayed in Figure 3, respectively.

While the performance of the PSTH does not give us a guaranteed lower bound on the achievable cross-entropy, it gives us a very optimistic estimate of the performance that can be achieved by a model which does not take spike history into account. We found that the PSTH yielded a significantly lower cross-entropy than an STM without history dependency (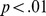), but not significantly lower than an STM which takes spike history into account (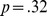 and 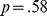, respectively; Figure 5C and D).

PSTHs for model cells were estimated from 1000 spike trains (sampling spike trains was necessary since the models depend on the spike history) using the same kernel as for the real cell. The variance explained (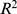) by the generalized linear model, quadratic model and STM was 0.15, 0.26, and 0.47 for the SA cell, and 0.19, 0.41, and 0.5 for the RA cell (Figure 3), respectively. Note that the explained variance depends heavily on the chosen kernel width and wider kernels would yield larger coefficients.

### How much data is enough?

The high firing rate of the cells and the resulting abundance of data allowed us to neglect regularization and overfitting issues. The training set contained on average about 25,000 spikes for SA cells and 6,700 spikes for RA cells. However, typically much less data is available.

To counter overfitting, different approaches to regularization can be taken. We already suggested reducing the number of parameters of the STM via factorization and parameter sharing (Equation 9). To get an idea of how the factored STM's performance behaves as a function of the available data, we artificially reduced the amount of data by randomly picking a subset of the 50 training trials. Of that subset, we used 50% for validation and 50% for optimization. During optimization, the performance on the validation set was tested every 5 iterations. If it decreased 50 times in a row, training was stopped and the parameters with the lowest validation error until then were kept. Other than early stopping, no other form of regularization was used. The test set was the same as the one used in Figure 5.

**Figure 6:**
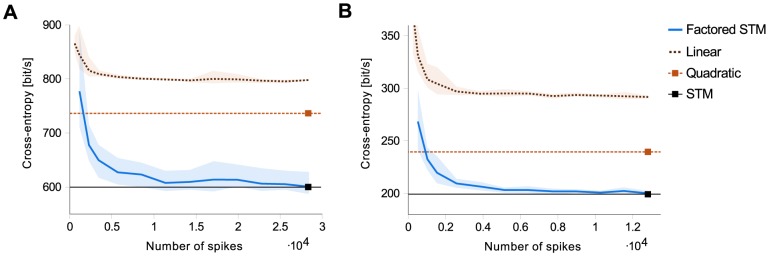
Performance as a function of available data. The factored STM was trained with different random subsets of the training trials and evaluated on all test trials for one SA cell (**A**) and one RA cell (**B**). The horizontal axis represents the number of spikes in the training set. Shown are the average performances (solid blue line) along with 90% confidence intervals (5th and 95th percentile). For comparison, we also show the performance of the linear model trained with different subsets of the data, the average performances of the non-factored STM, and the quadratic model trained on the entire training set. Note that the factored STM outperforms the generalized linear model even when only a small fraction of the dataset is used.

Figure 6 shows the performance of the factored STM for different amounts of spikes in the training set. The factored STM used 6 components and 5 quadratic features (246 parameters in total) for the SA cell, and 3 components and 5 quadratic features (198 parameters) for the RA cell. For comparison, we also plot the performance of a generalized linear model (111 parameters) trained with early stopping on a subset of the training data, as well as the performance of non-factored STMs (532 parameters and 400 parameters, respectively) and quadratic (156 parameters) models trained on the entire training set.

For the SA cell, the performance of the factored STM started to decrease more rapidly as soon as less than 5,000 spikes were present in the training set. However, even with 2,500 spikes the average performance was still much better than the performance of a quadratic model trained on the entire dataset. For the RA cell, the performance started to deteriorate at about 2,000 spikes. Note that the performance of the linear model worsened at a similar rate. Reducing the number of parameters further by using half the spike history or six instead of ten principal componets to represent the stimulus did not help to improve performance in the regime of few data points. The performance might however be improved by choosing suitable priors for the parameters, which we did not explore here.

Training with half the dataset of the RA cell (about 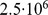 data points) on average took 9.4 minutes for the factored STM and 2.7 minutes for the linear model with parallelized implementations written in C++ when run on a machine with 12 Intel Xeon E5-2630 cores (2.3 GHz).

## Discussion

We have shown that a spike-triggered mixture model can lead to better performance than either linear or quadratic models, which we illustrated on the example of rat primary afferents. A possible explanation for the improved performance might be that our model can better cope with a cell's adaptation to the stimulus. Because the firing rate of our model is effectively a maximum over a number of quadratic models, the model is able to respond differently in different regions of the stimulus space. Our model may yield even bigger improvements when applied to cells higher up the hierarchy—such as cortical cells—where highly nonlinear dependencies on the stimulus are to be expected [21]. In particular, an interesting empirical question is whether STMs will be able to improve upon quadratic models in modeling complex cells [22]. As a generalization, the STM can capture the same kind of invariances that the quadratic model can capture, but in addition allows us to spend parameters in different ways by adding components instead of quadratic feature dimensions.

Here, we chose to give up on the constraint of convexity to be able to build a more flexible neuron model. In practice, non-convex or even multimodal likelihoods do not have to be an issue. Many local optima of the STM likelihood are created simply by permutations of the parameters of the different mixture components and are therefore unproblematic. We found that initializing mixture models with EM and fine-tuning with an off-the-shelf optimizer worked well for our data and the performance of the resulting model was stable across different intializations. The parameters of the factored variant of the STM (Equation 9) were randomly initialized and gave comparable results (Figure 6).

Alternatively, we could have used support vector machines, kernel logistic regression (KLR) [23] or other kernel based approaches [24] for gaining flexibility while retaining convexity. In KLR, the input to the sigmoid (Equation 2) determining the firing rate takes the form
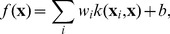
(12)where 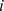 indexes training points 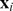 and 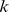 is a kernel measuring the similarity between stimuli or, more generally, inputs to the neuron. If a Gaussian RBF kernel is used, KLR becomes similar to an STM with all covariance matrices constrained to a multiple of the identity matrix and one mixture component placed on top of each data point (*cf.* Equation 8).

KLR is equivalent to a linear-nonlinear-Bernoulli model with a cleverly chosen feature space whose dimensionality grows with the number of data points. Hence, one advantage of KLR is that its objective function is convex. Advantages of a parametric model like the one presented in this paper are more readily interpretable parameters and lower computational costs when the number of training points is large. Ultimately, whether kernel based methods or a generative approach should be preferred presumably depends on whether one has a better intuition of what represents a good kernel for the input space, or a better intuition of what represents a good characterization of the spike-triggered distribution.

The idea of using spike-triggered distributions to construct and motivate neuron models is not new. However, most work in this direction has focused on spike-triggered averages and covariances [25]–[29]. Here we used mixtures of Gaussians and histograms to derive a new neuron model, but other distributions might work better in a different context and might be worth exploring.

Yet another related approach is to use feed-forward neural networks [30]–[32]. While standard feed-forward neural networks are in principle also able to represent arbitrarily complex stimulus-response relationships [33], one can hope to get away with fewer parameters, less data, or simpler optimization schemes when using a model tailored to the task at hand. In contrast to general nonlinear regression strategies, a generative approach can lead to much more problem-specific architectures and nonlinearities (Equations 8 and 9). Similar cascades of linear-nonlinear units have been proposed but motivated by physiological rather than statistical considerations [20], [34]–[36].

STMs can easily be extended to model populations of neurons similar to how GLMs are extended to populations by introducing coupling filters [5], [37]. Analogous to how we incorporated dependency on the spike history of a single neuron, a form for the dependency between neurons can also be motivated via a log-likelihood ratio for the distribution of cross-interspike intervals.

Code for fitting STMs is provided at http://bethgelab.org/code/theis2013a/.

## Supporting Information

Text S1 Gradients for the log-likelihoods of the STM and factored STM.
(PDF)
