# Supplementary material for: Beyond GLMs: A Generative Mixture Modeling Approach to Neural System Identification
Source: PLoS Comput Biol. 2013 Nov 21;9(11):e1003356. doi: 10.1371/journal.pcbi.1003356 (PMC3836720; doi:10.1371/journal.pcbi.1003356)
Supplement: Text S1 — Gradients for the log-likelihoods of the STM and factored STM. (PDF) [file pcbi.1003356.s001.pdf]

STM gradients

Here we derive the log-likelihood gradients for the parameters of the *STM* as defined by

$$p(y = 1 \mid \mathbf{x}, \tau) = \sigma(f(\mathbf{x}, \tau)),$$
$$f(\mathbf{x}, \tau) = \log \frac{\sum_k \alpha_{1k} \mathcal{N}(\mathbf{x}; \boldsymbol{\mu}_{1k}, \boldsymbol{\Sigma}_{1k})}{\sum_k \alpha_{0k} \mathcal{N}(\mathbf{x}; \boldsymbol{\mu}_{0k}, \boldsymbol{\Sigma}_{0k})} + \log \frac{h_{1\tau}}{h_{0\tau}} + \log \frac{\pi}{1-\pi},$$

where $\mathcal{N}(\mathbf{x}; \boldsymbol{\mu}, \boldsymbol{\Sigma})$ is the density of a Gaussian with mean $\boldsymbol{\mu}$ and covariance $\boldsymbol{\Sigma}$. We replace covariances $\boldsymbol{\Sigma}_{sk}$ by Cholesky factors of precision matrices,

$$\boldsymbol{\Sigma}_{sk} = \left(\mathbf{L}_{sk} \mathbf{L}_{sk}^\top\right)^{-1},$$

for $s \in \{0, 1\}$, where the $\mathbf{L}_{sk}$ are lower triangular, and optimize $\mathbf{L}_{sk}$ instead of $\boldsymbol{\Sigma}_{sk}$. For a single data point $(y, \mathbf{x}, \tau)$ and arbitrary parameter θ, the log-likelihood gradient is given by

$$\frac{\partial}{\partial \theta} \log p(y \mid \mathbf{x}, \tau) = (y - \sigma(f(\mathbf{x}, \tau))) \cdot \frac{\partial}{\partial \theta} f(\mathbf{x}, \tau).$$

For any of the mixture component parameters $\theta_{0k} \in \{\alpha_{0k}, \boldsymbol{\mu}_{0k}, \boldsymbol{\Sigma}_{0k}\}$ and $\theta_{1k} \in \{\alpha_{1k}, \boldsymbol{\mu}_{1k}, \boldsymbol{\Sigma}_{1k}\}$, we have

$$\frac{\partial}{\partial \theta_{1k}} f(\mathbf{x}, \tau) = \gamma_{1k}(\mathbf{x}) \frac{\partial}{\partial \theta_{1k}} \left(\log \alpha_{1k} + \log \mathcal{N}(\mathbf{x}; \boldsymbol{\mu}_{1k}, \boldsymbol{\Sigma}_{1k})\right),$$
$$\frac{\partial}{\partial \theta_{0k}} f(\mathbf{x}, \tau) = -\gamma_{0k}(\mathbf{x}) \frac{\partial}{\partial \theta_{0k}} \left(\log \alpha_{0k} + \log \mathcal{N}(\mathbf{x}; \boldsymbol{\mu}_{0k}, \boldsymbol{\Sigma}_{0k})\right).$$

where

$$\gamma_{sk}(\mathbf{x}) = \frac{\alpha_{sk} \mathcal{N}(\mathbf{x}; \boldsymbol{\mu}_{sk}, \boldsymbol{\Sigma}_{sk})}{\sum_{k'} \alpha_{sk'} \mathcal{N}(\mathbf{x}; \boldsymbol{\mu}_{sk'}, \boldsymbol{\Sigma}_{sk'})}.$$

Further, we have that

$$\frac{\partial}{\partial \alpha_{sk}} \log \alpha_{sk} = 1/\alpha_{sk},$$
$$\frac{\partial}{\partial \boldsymbol{\mu}_{sk}} \log \mathcal{N}(\mathbf{x}; \boldsymbol{\mu}_{sk}, \boldsymbol{\Sigma}_{sk}) = \mathbf{L}_{sk} \mathbf{L}_{sk}^\top (\mathbf{x} - \boldsymbol{\mu}_{sk}),$$
$$\frac{\partial}{\partial \mathbf{L}_{sk}} \log \mathcal{N}(\mathbf{x}; \boldsymbol{\mu}_{sk}, \boldsymbol{\Sigma}_{sk}) = \operatorname{diag}\left(1/L_{sk}^{ii}\right) - \operatorname{tril}\left((\mathbf{x} - \boldsymbol{\mu}_{sk})(\mathbf{x} - \boldsymbol{\mu}_{sk})^\top \mathbf{L}_{sk}\right),$$

where the L_{sk}^{ii} are the diagonal entries of $\mathbf{L}_{sk}$, $\operatorname{diag}\left(1/L_{sk}^{ii}\right)$ is the diagonal matrix whose diagonal entries are $1/L_{sk}^{ii}$, and $\operatorname{tril}(\mathbf{A})$ is the lower triangular part of the matrix $\mathbf{A}$.

Finally, the gradient of $h_{1\tau}$ is given by

$$\frac{\partial}{\partial h_{1\tau}} f(\mathbf{x}, \tau) = \frac{1}{h_{1\tau}}.$$

The parameters $h_{0\tau}$ and the parameter π are redundant and we did not optimize them.

Factored STM gradients

Here we derive the log-likelihood gradients for the parameters of the *factored STM* as defined by

$$p(y=1 \mid \mathbf{x},\mathbf{z}) = \sigma\left(f(\mathbf{x},\mathbf{y})\right),$$

$$f(\mathbf{x},\mathbf{z}) = \log \sum_k \exp\left(f_k(\mathbf{x})\right) + \mathbf{v}^\top \mathbf{z},$$

$$f_k(\mathbf{x}) = \sum_{n=1}^{N} \beta_{kn} (\mathbf{u}_n^\top \mathbf{x})^2 + \mathbf{w}_k^\top \mathbf{x} + a_k.$$

For a single data point $(y,\mathbf{x},\mathbf{z})$ and arbitrary parameter θ, the log-likelihood gradient is given by

$$\frac{\partial}{\partial \theta} \log p(y \mid \mathbf{x},\mathbf{z}) = (y - \sigma(f(\mathbf{x},\mathbf{z}))) \cdot \frac{\partial}{\partial \theta} f(\mathbf{x},\mathbf{z}).$$

If θ_k is any parameter of component k, we have

$$\frac{\partial}{\partial \theta_k} f(\mathbf{x},\mathbf{z}) = \gamma_k(\mathbf{x}) \frac{\partial}{\partial \theta_k} f_k(\mathbf{x})$$

where

$$\gamma_k(\mathbf{x}) = \frac{\exp\left(\mathbf{x}^\top \mathbf{K}_k \mathbf{x} + \mathbf{w}_k^\top \mathbf{x} + a_k\right)}{\sum_{k'} \exp\left(\mathbf{x}^\top \mathbf{K}_{k'} \mathbf{x} + \mathbf{w}_{k'}^\top \mathbf{x} + a_{k'}\right)}.$$

For the individual parameters, we get

$$\frac{\partial}{\partial \beta_{kn}} f_k(\mathbf{x}) = (\mathbf{u}_n^\top \mathbf{x})^2,$$

$$\frac{\partial}{\partial \mathbf{w}_k} f_k(\mathbf{x}) = \mathbf{x},$$

$$\frac{\partial}{\partial a_k} f_k(\mathbf{x}) = 1.$$

Finally, for $\mathbf{u}_n$ and $\mathbf{v}$, we have

$$\frac{\partial}{\partial \mathbf{u}_n} f(\mathbf{x},\mathbf{z}) = 2 \sum_k \gamma_k(\mathbf{x}) \beta_{kn} (\mathbf{u}_n^\top \mathbf{x}) \mathbf{x},$$

$$\frac{\partial}{\partial \mathbf{v}} f(\mathbf{x},\mathbf{z}) = \mathbf{z}.$$
